# 

**Published:** 1914-10-24

**Authors:** 


### CONTENTS.

| | PAGE |
|---|---|
| **The Cumberland Infirmary, Carlisle** ... ... | 77 |
| **The Salaries of Deputising Medical Officers** ... | 78 |
| **Hospital and Institutional News** ... ... ... .. | 79 |

The Cumberland Infirmary, Carlisle—The Health of the German Armies—" Utterly Inadequate " Hospital Accommodation — Mr. Howard Collins' Death—The Poor Law and the Red Cross—Dr. Keats Awarded Damages for Libel —Hospital Ships for the Indian Troops—Accommodation at Netley for Indian Wounded —Supplies of Vaccine for the Armies in France—The Queen's Canadian Military Hospital—The Staff Trouble at Lambeth Infirmary—Special Emoluments for those Remaining —Mentioned in Dispatches—Mentioned in Dispatches: Royal Army Medical Corps—Hospitals and Insurance in 1915—Effect of Exodus from East Coast on Hospital Work—The Declining Vogue of the Operation —The Evolution of the Hospital Ship—Maternity Safeguards in War-Time—Hospital Legacies in Time of War—A Hospital Compared to Black Hole of Calcutta—A Medical Officers' Responsibility—Claims of Civil Population at Cardiff—This Week's Drug Market.

### CONTENTS—(*continued*).

PAGE

**My Experiences in a Field Ambulance** ... ... 85
By an Officer of the British Expeditionary Force.

**The Death of Mr. Howard J. Collins** ... ... 87
A Memoir by One Who Knew Him Well.

**A Standard Method for Examining Water** ... 88
The Report of the Recent Committee.

**British and Belgian Wounded at Cardiff** ... 89
Scenes in the Busiest Week of the War.

**Hospitals and the War** ... ... ... ... ... 90
Bath : The Medical Mayor's Efforts—Birmingham : Wounded from Malines—Bristol—1,000-Bed Hospital at Cambridge—Belgians' Stories at Chatham — Colchester — Doncaster : The Mansion House Hospital—Gravesend : The Overflow from Dover—Hastings : Influx of Wounded and Refugees—Leicester : General Ford's Visit — Norwich : Tents for the Wounded—Rochester—Sunderland : The First Convoy—Swansea : Active Service List.

PAGE

**Round the World** ... ... ... ... ... 94
Fellow-Workers and the Roll of Honour.

**The Welsh War Hospital, Netley** ... ... ... 95
The Movable Hospital to be Opened Next Week.

**"A Good Clinician"** ... ... ... ... ... ... 96

**The Ambulance Dog in War** ... ... ... 96

**Medical Appointments** ... ... ... ... ... 96

**Buildings Contemplated** ... ... ... ... 98

**Gifts and Bequests** ... ... ... ... ... 98

**The Book Market** ... ... ... ... ... 98

**The Institutional Worker** ... ... (Suppl.) 1
Electrical Cooking for Hospitals.
Questions for October.
Questions Invited.
The Sick and in Need.
Employment and Training.
War Matters.

